# Feasibility and applicability of self-sampling based online cervical cancer screening: findings from the China online cervical cancer screening trial

**DOI:** 10.1186/s13027-024-00583-6

**Published:** 2024-04-25

**Authors:** Yi Zhang, Hui Du, Chun Wang, Xia Huang, Xinfeng Qu, Ruifang Wu

**Affiliations:** 1 Center of Obstetrics and Gynecology, Peking University Shenzhen Hospital, Shenzhen, 518036 People’s Republic of China; 2 Institute of Obstetrics and Gynecology, Shenzhen PKU-HKUST Medical Center, Shenzhen, 518036 People’s Republic of China; 3 Shenzhen Key Laboratory On Technology for Early Diagnosis of Major Gynecologic Diseases, Shenzhen, 518036 People’s Republic of China

**Keywords:** Online, Cervical cancer screening, Self-registration, Self-sampling, HPV testing

## Abstract

**Objective:**

This study aims to evaluate the feasibility and applicability of an online cervical cancer screening program using a website as the public platform and self-collected HPV testing as the primary screening method.

**Methods:**

A website (mcareu.com) was developed to facilitate the online cervical cancer screening program by Peking University Shenzhen Hospital (PUSH). Women in Shenzhen could register for participation on the website by providing essential demographic data. Sampling kits and specimens were delivered through regular logistics. Eligible women collected vaginal samples by themselves using the provided kits and in referring of the graphic guidance. The specimens were tested for HPV at PUSH or a reference lab, and the results were accessible on the website through participants' personal accounts. Participants who tested positive for high-risk HPV were scheduled for colposcopy and biopsies. The demographic and social background data of the eligible participants were analyzed to evaluate the feasibility and applicability of the online screening approach.

**Results:**

A total of 1712 applicants registered for participation, with 99.9% (1710/1712) completing registration with full data. The analysis included 1560 applicants aged 30–59, with an average age of 41.1 (± 7.6) years. Among them, 83.3% (1299/1560) provided self-collected samples for testing. Age-group analysis revealed an overall sample provision rate (SPR) exceeded 80% in all age groups. A significant difference in SPR was observed only between the 30–34 and 45–49 age groups (*p* < 0.05), while no significant differences were found among other age groups. 99.7% of the samples were tested qualified, and there was no significant difference in sampling failure rate among age groups. Analysis of demographic and social elements showed no significant impact on the rates of sample provision among groups in most of the social elements but the medical insurance and the monthly family-incomes.

**Conclusion:**

The findings demonstrate that online cervical cancer screening is reliable for self-registration, self-sampling, and self-ordering for specimen transportation. It is suitable for women of all ages needing to be screened, irrespective of social elements, and effectively facilitates screening for women with limited access to medical resources. Therefore, online screening holds promise as an effective approach to increase screening coverage.

## Introduction

Cervical cancer is the fourth leading cause of cancer morbidity and mortality in women worldwide, affecting at least 50% of women during their lifetime [1]. Almost all (99%) women with cervical cancer were linked to infection with high-risk human papillomaviruses (hrHPV), an extremely common virus transmitted through sexual contact. On the other hand, only a small part of hrHPV infections persist and develop into high grade cervical interepithelial neoplasia (CIN) which may further progress to cervical invasive cancers if left untreated [2]. Cervical cancer is preventable via early detection through screening and proper treatment of the CIN-2/3s [3].

In July 2020, World Health Organization (WHO) released “global strategy to accelerate elimination of cervical cancer as a public health problem” [4], in which the elimination of cervical cancer was goaled at an incidence below 4 per 100,000 women. WHO also set “90-70-90” global objectives for year 2030, which refer to 90% of girls to be vaccinated with HPV vaccine by 15 years of age, 70% of women to be screened by age of 35 and 45 years, and 90% of women with cervical cancers and precancers to be properly treated, respectively, by year of 2030. Those objectives must be maintained for decade to reach the goal of cervical cancer elimination [4].

China has the second largest number of annual cervical cancer cases in the world, with 110,000 occurrences and 59,000 death [1]. As a country with the biggest population and heaviest burdens of cervical cancer in the world, China must play an important role in achieving the global goal of eliminating this disease. Since late twentieth century, Chinese scholars have carried out numbers of studies in validation of cervical cancer prevention technologies and the governments have heavily granted in cervical cancer control [5]. However, China as many of the world low-and-middle-income-countries (LMICs) still face challenges in having cervical cancer screening reach the most vulnerable women, which will obviously become the an unignorable obstacle for China to achieve satisfied screening coverage set by the WHO. One survey showed that, in average, only 25.7% (30.0% in urban area and 22.6% in rural area) of women aged from 20 to 64 in China were screened by 2015 [5, 6], leaving a big gap between the current coverage and the targeted 70% to be filled in 15 years. It is obvious that, in addition to affordable technologies, new service models are equally important and urgently required for cervical cancer prevention in LMICs including China to ensure WHO objectives be achieved within the designated timeline.

Nowadays, HPV testing has been widely accepted as the most effective primary screening method for cervical cancer in LMICs. Comparing with Cytology, HPV testing has higher sensitivity for detecting of CIN2+ and higher negative predictive value, which prolongs the screening intervals [7], Prolonged screening interval with HPV testing makes HPV testing the most cost-effective technology when compared with cytology in terms of the required number of screening runs that guarantee satisfied control of cervical cancer throughout a woman's lifetime.

Our previous trials had validated that self-collected vaginal samples were equal sensitive with physician-collected cervical samples, with just slight but acceptable loss of specificity, when tested with PCR based HPV assays [8], no matter applying the samples in liquid (PreservCyt) or on solid cards containing reagents that can lyse cells and protect DNA [9]. In consideration of that self-sampling does not just change the way of sampling but also the manner of services, we developed community-participatory model for cervical cancer screening in collaboration with Preventive Oncology International (POI) [10], which provided innovative perspectives for new studies in service models for expanding screening coverage with self-collected HPV testing as the primary screening.

However, in addition to service models, many cognitive barriers also hinder women from participation in the screening programs, such as inconveniences in clinic appointments or clinic visits, poor knowledgeability on the importance of screening, embarrassment for physician sampling [11], and psychological resistance to detection of sexual transformation diseases. An internet-based public platform is undoubtedly an effective resource to provide easily accessible information that helps to rectify the public cognitions and to encourage women to participate in cervical cancers screening programs. Another important barrier that could be the most speed-limit procedure in self-sampling based mass screening is the screening registration, which conventionally be conducted by medical providers. An internet-based platform that enables self-registration by the participants themselves will be the solution, which has been proven by our previous study that online self-registration with essential demographic data makes no error [12]. Besides, we need an internet-based database to store screening data, identify women who need screening for proper intervals, program screening activities, and plan new screening programs [12], since HPV-testing-based screening just need a woman to be screened in an interval of 3–5 years.

Many studies have reported and summarized the validity of internet based self-sampling screening models for sexually transmitted infections (STIs) [13–16]. Variety of ways to improve screening participation and satisfaction based on a concept of telemedicine have been tried in variety of studies, including calling for participants at proper ages through text messages [17], sending weblink via text message to recruit participants, or online questionnaire to investigate participants’ experience and opinions about screening [18]. Although those trials were all focused on increasing coverage for hospital-centered screening projects, they provided evidence that gives us confidence in the potential of the internet to play important roles in facilitating community screening based on self-sampling. These roles include public education, screening registration, identifying individuals in need of screening, and sharing data records among all involved parties.

The objective of this study is to assess the feasibility of establishing a website as a public platform for cervical cancer prevention and control. The website will be used to facilitate an online cervical cancer screening program with self-sampling as the primary testing method.

## Methods

### The website for cervical cancer screening

A website http://www.mcareu.com was developed by our team with patented copyright for cervical cancer screening provided by Peking University Shenzhen Hospital (PUSH). The website was supervised under the certificated network security level protection of PUSH. The initial version of the website was developed to include modules for public education, self- registration, self-sampling, and result reporting, which are purposed to provide women with the screening project information and the background information on cervical cancer prevention, to facilitate women to complete screening registration by inputting personal data and sign informed consent form online, to facilitate eligible women to get sampler, collect sample for themselves, and apply logistic for shipment of the samples, and to enable women to check their primary testing results, respectively. The website can be log in on computers or mobile devices. Although accessible to all women in China, the project was only open for the participation of women who lived in Shenzhen.

### The project

After developing the website and completing its testing, we organized an online cervical cancer screening project conducted in 2 stages from March 2013 to May 2015 and August 2019 to June 2020, in which two deferent HPV assays and the liquid-based and card-based specimen processing media were adopted respectively in the first and second stages. The primary objectives of the project are to test (1) whether it is reliable to have none-professionally trained women to input their own data (self-data-input), (2) if women can collect qualified vaginal sample for themselves in referring to a graphic sampling guidance without instruction of on-site professionals, and (3) whether women can correctly follow instructions to order sample shipment to the lab. The project was divided into two stages because the first stage was approved to provide evidence for the safety and applicability of the website. The second stage was conducted after the website was officially approved for public services. When the second stage of the project was launched, an online questionnaire on participants’ background information was added to collect data for analysis of (the secondary objectives) (1) the willingness of the participants to respond a questionnaire for background information, and (2) the potential impacts of the background social elements on the online participation, in addition of the primary objectives of the study.

The program was designed to screen women who was eligible and registered successfully, with self-collected HPV testing as the primary screening test, and to biopsy all women who were primarily tested positive of hrHPV and returned for diagnostics. All those procedures were free of charge to eligible women. Anyone who was diagnosed as grade 2 cervical intraepithelial neoplasia and above (CIN2+) were referred to PUSH or other qualified hospitals for treatment. This study was approved by the Ethics Committee of the Peking University Shenzhen Hospital (G2014-1).

### Participants

With referring the project notification and the background information on cervical cancer prevention that was releases on the website, women who were living in Shenzhen could register for participation in the project by inputting their personal data online. Women would be eligible for participation and successful for registration if they were 25–59 (Stage 1) or 30–59 (Stage 2) years of age, none-pregnant, without history of hysterectomy or pelvic radiative therapy, willing to participate by signing an online version of informed consent form. Women were informed that they could withdrew from their participation at any time during the procedures without any impacts on their further medical cares. Women aged 25–29 years of age were included in the first stage of the project because (1) the screening guideline at that time allowed provision cervical cancers screening for women at that age per their willingness, and (2) we tried to include some younger women to test whether internet facilitation worked equal to women aged 55–59 as to young people aged to 25–29. This age group were not targeted in the second stage when we found no significant difference between age 25–29 and 55–59 in errors from self-input data.

### Participation procedures

The website was accessible for registration during the program stages. With accessibility control mechanism, only women who were living in Shenzhen can register for participation. This limit was reset in consideration of the feasibility of positive triages. No advertisement on public media was released other than the announcement of the program on the website, and no offline contact such as test message or phone call was given to the community women. With this regard, community women could just get known about the project via searching websites with cervical cancer screening related words and oral communications. In this way, we tried to keep most of the participants be screened per their original willingness based on their recognition on cervical cancer prevention without impacts from information resources other than the website. To keep women’s willingness for participation be based on same level of website information, no offline consultation was available before screening results were reported.

Successful registration for participation needed a woman to visit the website (mcareu.com) to “apply for screening” as an applicant. And the online version of informed consent form needed to be reviewed and signed by applicants by checking the reset statement in Chinese, which meant in English as: “I have read the contents of the informed consent and I agree to participate with full understanding of all the items.” The applicant would then get into a field for her to input her essential demographic information (EDI) required for screening tests. Then the registration module of the system would automatically match the applicant’s information with the eligibility criteria that had been reset into the system. Applicants who were eligible for participation would get a reminder for successful registration immediately after the EDI was input, while ineligible applicants would be informed for unsuccessful registration.

EDI required for registration included the name, State ID, living address, and contact details (cellphone number) of the applicants. Only the applicant who provided full items of the required EDI could be enrolled in the screening program as a participant (the participant). A barcode would be created for each of the participant, which was unique to a specific participant and used for identification of the participant in the whole program procedures. All participants could receive a sampling kit shipped to their registered addresses via regular logistics services.

In the second stage of the program, a questionnaire about the social background were provided to the participants to be completed per their willingness. The questionnaires included several questions including cervical cancer screening history, health insurance status, educational background, monthly family income, age of first sex, number of sexual partners, and smoking history, et al.

**Fig. 1:**
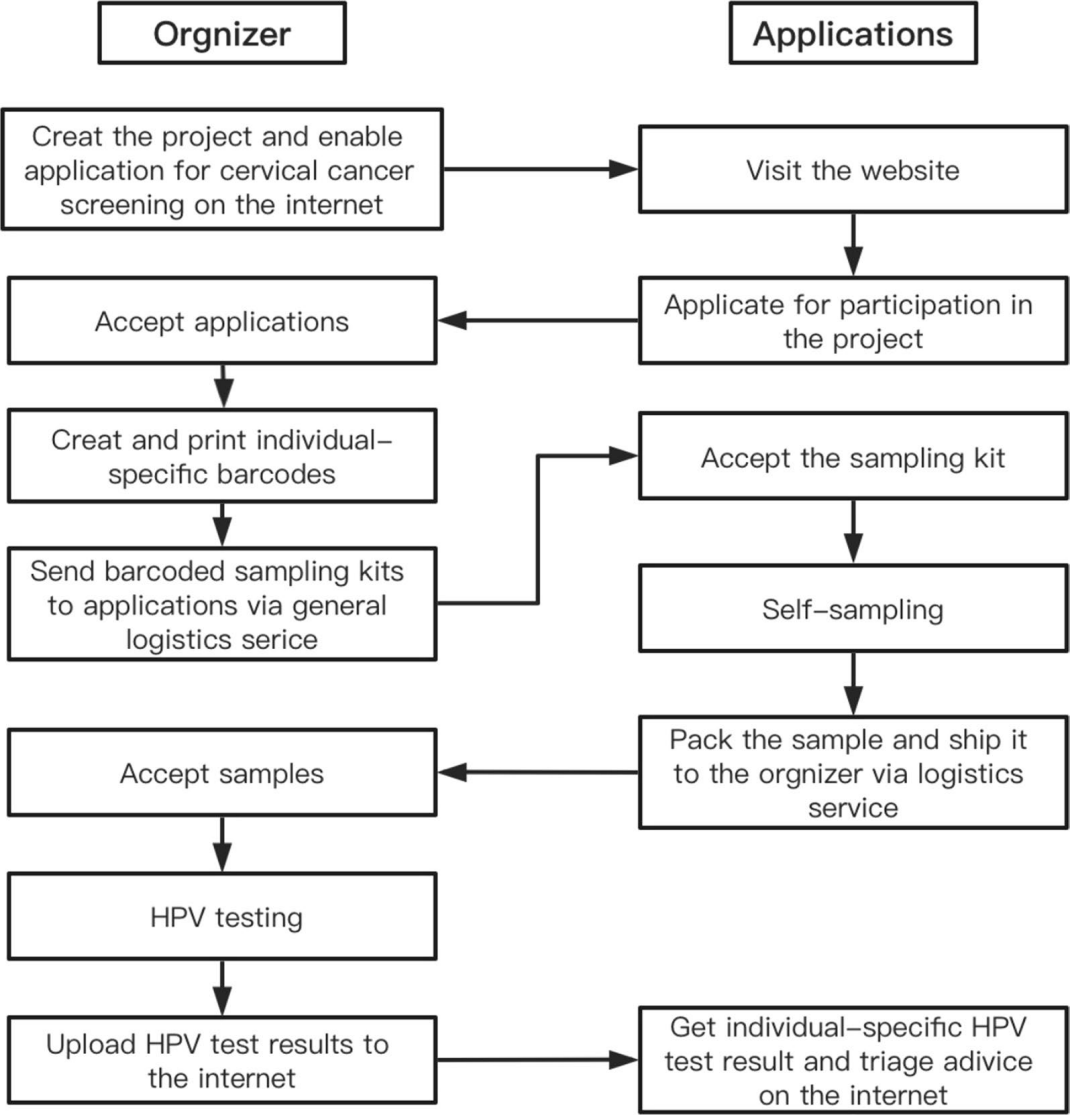
Flowchart of the project

When the screening events were launched, a sampling kit was provided to each of the participants, which contains a sampling brush, a piece of self-sampling guidance in graphics, an envelope, and a liquid-based specimen vial (for Stage 1) or a specimen processing card (for Stage 2). Participants were instructed to watch a video guidance on the website to learn how to get sample. A graphic guidance was available for each of the participants when they collected sample for themselves. After sample collection, participant (in the first stage) who used a kit containing liquid-based vial needed to put the brush with specimens into the vial and keep the brush head in the vial by breaking the handle off, while that (in the second stage) who used a kit containing a card needed to apply the specimen on the brush head onto the card. With specimen being put into the envelop, participants can apply logistics service on the website to ship the specimen to PUSH for hrHPV testing in the Obstetrics and Gynecology Laboratory of PUSH (Stage 1) or BGI lab under supervision of the investigators (Stage 2). No professional instruction was available online and offline during the whole sampling procedures (Fig. 1).

### HPV testing

Samples from Stage 1 were tested for hrHPV on Cobas4800 assay (Roche, USA), and the samples from Stage 2 were tested on SeqHPV assay (BGI, Shenzhen, China). Cobas4800 is a multi-PCR based HPV assay detecting 14 types of hrHPV in three channels respectively for HPV16, HPV18, and 12 other HPV types in pool (pooled 12-HPV) (HPV-31, -33, -35, -39, -45, -51, -52, -56, -58, -59, -66, and -68). SeqHPV is a high-throughput assay based on multiplex PCR for DNA extraction and amplification and next generation sequencing for HPV genotyping. It is configured to detect and report 14 hrHPV types (HPV-16, -18, -31, -33, -35, -39, -45, -51, -52, -56, -58, -59, -66 and -68) individually. It had been validated in a cross-sectional clinic trial to have the same sensitivity and specificity with Cobas4800 in detection CIN2+ and CIN3+ lesion using both physician- and self-collected specimens [20]. To conduct comparative analysis, we categorized hrHPV positive results to be three hierarchies as HPV16, HPV18, and 12-HPV in-pool, which refers to positive of HPV16 only and multi-type of hrHPV including HPV16, positive of HPV18 and multi-type hrHPV including HPV18 but excluding HPV16, and positive of single-type of hrHPV other than HPV16 or 18 and multi-type of hrHPV excluding HPV16 and/or 18, respectively [19].

### Management of the positives

HPV testing results were uploaded on to the website, which were individual-specific for access by the participants with a validate password and username or ID number. Participants who tested positive of hrHPV (the positives) could schedule a diagnostic visit to PUSH on the website. Offline contacts through phone call were given to the positives participants who did not return for triage in one month after the results were reported online, to encourage them to visit the hospital for management.

Colposcopy and biopsies were performed on all positives returned for management, which were conducted by trained clinicians from PUSH following a POI-protocol developed by Preventive Oncology International (POI). According to that protocol, colposcopy-directed biopsy was taken at all sites with visible lesion and random biopsies were taken at all sites without visible lesion in any or all the 4 quadrants plus endocervical curettage (ECC) [20]. All the biopsied specimens were processed in PUSH lab and analyzed by a gynecological pathologist from PUSH, who was blind of HPV genotype information and the colposcopy impressions. Pathology diagnoses were reported according to the 2014 WHO Classification of Female Genital Tumors [21].

### Data analysis

To evaluate the effectiveness of the internet platform in self-sampling based cervical cancer screening program, analysis of the data will be focused on: (1) the rate of qualified registration data among the applicants, (2) the rate of sample provision among those who got the sampler, (3) the rate of qualifies samples, (4) the rate of questionnaire response among enrolled participants, and (5) potential elements that impact on the online screening. To analyze the composition of the participants in internet screening, the demographic and background data of the participants and the clinical records were compared by grouping the participants in variety of dimensions. Univariate regression analysis was conducted to identify the association of demographic and historical details with the participation and sampling rate. The chi-square test was used to conduct comparative analyses. SPSS v.26.0 software (IBM, Armonk, NY, USA) was used for all data analysis in this study with the significance level to be *p* < 0.05.

## Results

**Fig. 2:**
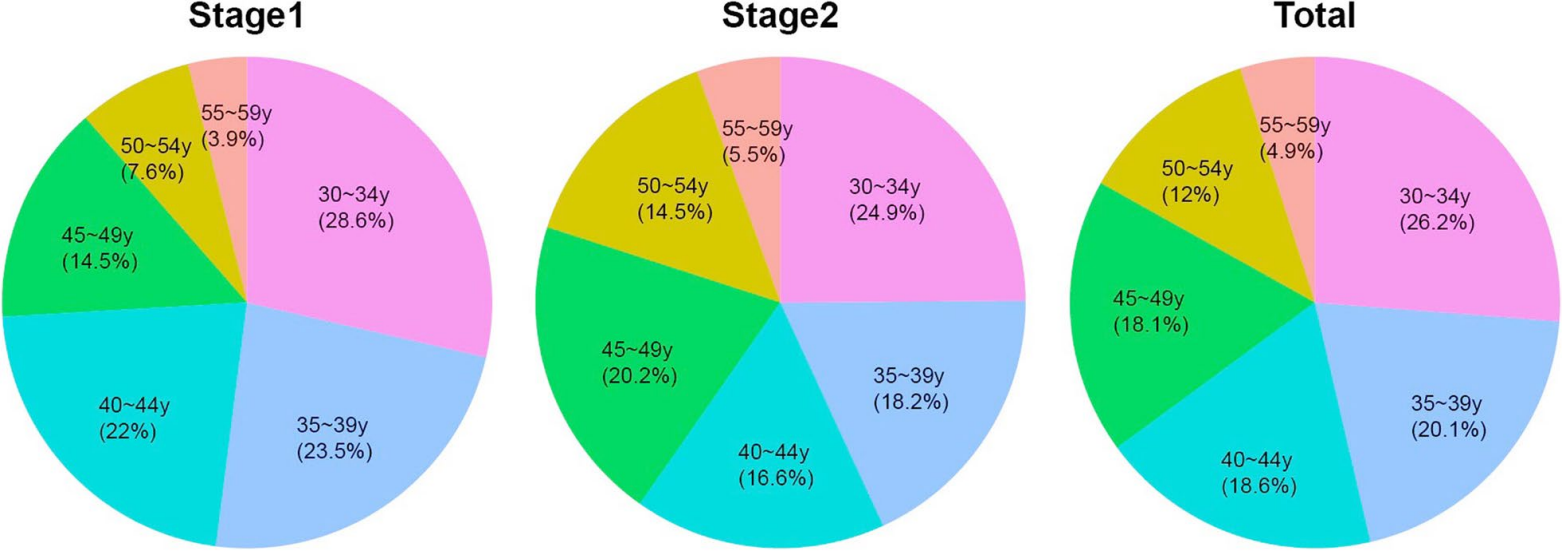
Age distribution of the full-data applicants from Stage1, Stage2, or the total of the 2 stages

A total of 1712 applicants (719 from Stage 1, and 993 from stage 2) registered for self-sampling screening on the website and 99.9% (1710/1712) of them completed registration through inputting the full data required for registration (the full-data applicants), with only 2 from Stage 1 who were not able to complete the registration. In order to analyze the age-composition of the full-data applicants, we combined the applicants from the 2 stages at age of 30–59 years, without including 150 participants aged 25–29 years, making 1560 full-data applicants (567 from Stage 1 and 993 from Stage 2) for analysis. The average age of the full-data applicants for analysis was 41.1(± 7.6) years, and 26.22% (409/1560) of them were at age 30–34, 20.06% (313/1560) at age 35–39, 18.59% (291/1560) at age 40–44, 18.21% (284/1560) at age 45–49, 11.99% (187/1560) at age 50–54, and 4.94% (77/1560) were at age 55–59 of years (Fig. 2). The average age of women from the Stage 2 (41.8(± 7.8)) were higher than women from Stage 1(39.8(± 7.2)), for there were more women among age 45–59 in Stage 2 (Table 1).

**Table 1:** Details of application, participation, sample provision and qualified samples by age group from 30–59 (n = 1561)

	Total	30–34	35–39	40–44	45–49	50–54	55–59
Application	1561	409	313	291	284	187	77
Full data Applicants	1560 (99.9)	409 (100)	313 (100)	290 (99.6)	284 (100)	187 (100)	77 (100)
Samples^*^	1299 (83.3)	328 (80.2)^*^	255 (81.5)	236 (81.4)	253 (89.1)^*^	161 (86.1)	66 (85.7)
Qualified Samples	1295 (99.7)	327 (99.7)	255 (100)	234 (99.2)	252 (99.6)	161 (100)	66 (100)

^*^Significant difference between group “30–34” and group “45–49” (*p* < 0.05), no difference among other groups

Eighty-three-point-three percent (83.3%, 1299/1560) of the full-data applicants for analysis had provided self-collected samples via logistic services in the requires term and packages. The overall sample provision rate (SPR) was over than 80% in all age groups with age-group specific SPR to be 80.2% (328/409) at age of 30–34, 81.5% (255/313) at age of 35–39, 81.4% (236/290) at age of 40–44, 89.1% (253/284) at age 45–49, 86.1% (161/187) at age of 50–54, and 85.7% (66/77) at age of 55–59. Significant difference on SPR was only found between age groups of 30–34 and 45–49 (*p* < 0.05), but not among other age groups (Table 1). Further analysis shows that this overall difference is due the significant difference between the 2 age groups in Stage 2. Analysis per stages shows that Stage 2 achieved a higher SPR than Stage 1 with significant difference (*p* = 0.046). Those results suggests that online screening is suitable to all age of women with age 30–59, the neediest age for cervical cancer screening.

Of the 1299 samples self-collected by women at variety of ages without instruction or help of the onsite providers, 99.7% were tested qualified for test. No significant difference was found among age groups in sampling failure rate, although group 40–44 got the highest testing failure rate (Table 1). This results indicate that self-sampling is applicable for all ages of women.

Of the 993 enrolled participants in Stage 2, 97.3% (966/993) fulfilled the questionnaire on the website when they registered for participation, and 919 of them submitted complete questionnaire with the overall questionnaire completion rate (QCR) of 95.1% (919/966). The questionnaire fulfill rate was observed to be the lowest in age group of 35–39 and highest in age group of 50–54, however, no significant difference among different age groups in the QCR (Table 2). Since not all the enrolled participants in Stage 2 who fulfilled the questionnaire gave answers for all the questions, we tried to analyze the result from each question based on the number of participants who provided answers for that specific question. The statistical results of the questionnaire were shown in Table 3. When we grouped the participants based on various social elements that could potentially impact their participation in online screening, we find no significant difference among groups in most of the social elements but the medical insurance and the monthly family-incomes, which shows that the SPR of participants with medical insurance was significantly higher than those without medical insurance, and the SPR of those with a monthly family-income of over than RMB10,000 was significantly lower than those with monthly family-income of RMB5000–10,000 and those with monthly family-income less than RMB5000 (*p* < 0.05) (Table 3). Univariable regressive analysis showed that age of first sex, number of sexual partners, smoking et al*.* were not significantly associated with cervical cancer screening history and the participation of the online screening.

**Table 2:** Questionnaire responding and complication rate by age group (stage 2, n = 993)

	Total	30–34	35–39	40–44	45–49	50–54	55–59
Applicants	993	247	181	165	201	144	55
Responded*	966 (97.3)	240 (97.2)	170 (93.9)^*^	159 (96.4)	200 (99.5)	144 (100)	53 (96.4)
Completed	919 (95.1)	230 (95.8)	166 (97.6)	151 (95)	185 (92.5)	135 (93.8)	52 (98.1)

^*^Significant lower than “45–49”and “50–54” (*p* < 0.05)

**Table 3:** Categorization of the 966 questionnaire respondents from stage 2 (n = 966)

Elements	Category	n (%)	Sampling rate
Screening history (n = 955)	Never screened before	452 (47.3)	83.4 (377/452)
	Previously screened	503 (52.7)	87.1 (438/503)
Health insurance (n = 966)	With insurance	876 (90.7)	86.2 (755/876)
	Without insurance	90 (9.3)	74.4 (67/90)^*^
Education (n = 963)	Less than middle school	45 (4.7)	86.7 (39/45)
	Middle school	214 (22.2)	84.6 (181/214)
	College degrees	704 (73.1)	85.2 (600/704)
Monthly family income (n = 954)	Less than 5000 yuan	191 (20.0)	88.5 (169/191)
	5000 to 10,000 yuan	541 (56.7)	89.8 (486/541)
	10,000 yuan or more	222 (23.3)	72.1 (160/222)^*^
Age of first sex (n = 929)	< 20 years	180 (19.4)	84.4 (152/180)
	≥ 20 years^*^	749 (80.6)	84.8 (635/749)
Number of sexual partners (n = 875)	1	842 (96.2)	85.7 (722/842)
	≥ 2	33 (3.8)	78.8 (26/33)
Smoking (n = 927)	Yes	16 (1.7)	81.3 (13/16)
	No	911 (98.2)	84.6 (771/911)

^*^Significant difference with the other group(s) (*p* < 0.05)

The hrHPV positive rate of the 1295 women with HPV testing results was 16.2% (210/1295). Of the 210 positives, only 123 (58.6%, 123/210) returned for colposcopy and triages, with a call-back rate of 58.6% (Table 4). However, this rate couldn’t demonstrate whether the internet facilitation increases the diagnostic rate for the primary positive cases, because the website had no module enabling providers to input positive management data and we allow the enrolled participants to search for medical serviced in nearby medical services if they were screened positive of hrHPV.

**Table 4:** Screening outcomes of the applicants with qualified HPV tests (n = 1295)

Characteristics	Applicants (n (%))
Qualified samples	1295
**hrHPV-positive**	210 (16.2%)
HPV16	30 (2.3%)
HPV18	11 (0.8%)
Pooled 12-HPV	169 (13.0%)
**Histology**	123 (58.6%)
Non-CIN	90 (73.2%)
CIN1	17 (13.8%)
CIN2+	16 (13.0%)

## Discussion

In this study, we evaluated the feasibility and applicability of internet-facilitated online screening for cervical cancer, through analyzing the data for self-registration, self-sampling, and self-order for specimen shipment. The results showed that online screening for cervical cancer using self-collected HPV testing as the primary screening, along with a professionally designed website as the data platform, is feasible, reliable, and practical. Additionally, online screening is suitable to all women at age 30–59 as well as those at age 25–29. Furthermore, online screening can effectively reach women who had never been screened for cervical cancer. These results are supported by the facts in our study that 99.9% applicants could complete self-registration online with correct data confirmed by the auto-checking of the website, 83.3% of the enrolled participants provided their samples with the SPRs exceeding 80% across all age groups, 99.7% of the samples were self-collected by the participants correctly in referring the online or offline graphic sampling guidance, and 47.3% of the participants who responded the question about cervical cancer screening history had never been screened for cervical cancer before.

Most encouragingly, 120,099 people from the overall country visited the website when it was put online for the Stage 1 screening during March 2013 to May 2015. If the screening program were accessible to all of them and in consideration of the rates in our study, we could potentially made near one hundred thousand (99,642 = 120,099 * 99.9% * 83.3% * 99.7%) women to be screened. Compared to the previous studies with tele-medicine model which recruited participants by sending information to target women and enroll them offline [18, 22], online screening under internet facilitation can recruit and enroll more women with comparative higher cost-effectiveness. The 80% of SPR at all ages achieved by our study also evidenced that, if endorsed by governments, community authorities, religion groups, or any organization with good public respects and trusts, along with public education efforts, online screening has the potential to cover more than 80% of eligible women in a specific region or city for cervical cancer screening. This level of coverage would align with the WHO's screening target for 2030.

Our study demonstrates that online screening based on a professionally designed website enables cervical cancer screening to be conducted without involvement of medical providers in the registration and sampling procedure, which is vital to expanding screening coverage and promoting the procedure for self-registration. The 83.3% of the overall SPR and the 80% of age specific SPRs in all age groups served as strong evidence of the public's acceptance to online screening and self-sampling. This is in sharp contrast to certain studies, in which the sampling kits were mailed directly to the potential participants for self-sampling without prior registration, and the sample return rate was less than 30% [22, 23].

With the 99.7% of qualified samples in tests, our study also demonstrates that a graphic sampling guidance, no matter printed on paper for offline or online reference, can guide the women to collect vaginal samples for themselves correctly without needing many onsite providers to provide guidance for self-sampling, to confirm sample-quality, and to preserve the samples, which was the way in many studies when self-sampling was adopted [24–26]. Realistically online screening would have no advantage to the offline ones if the online platform could not guarantee the reliability of the self-registration and self-sampling to save personals for data inputs on those procedures.

To give answers to a question for whether any of demographic and social elements can result in any difference among screening participation, we conducted an analysis through grouping and comparing participants based on their responses to the questionnaire survey. The results of this analysis were highly positive, indicating that both online and offline cervical cancer screening are widely accepted by women, regardless of their demographic and social backgrounds. Lower SPRs were observed in women without medical insurance and those with higher family incomes, for which we need evidence to explain why. However, the fact that more than 47.3% (452/995) of the participants in the second screening stage had never been screened before suggests that online screening can effectively reach women who have limited access to cervical cancer screening services. This is crucial for increasing the overall screening coverage.

The convenience and privacy of online screening had been widely recognized. A lot of studies have confirmed that the sensitivity of self-sampling is comparable to that of physician-sampling in detecting CIN2+ when tested on PRC-based HPV assays [27], and self-sampling is highly accepted by women at different ages, of different races, and with different nationalities [28–31]. Some study indicated that the accuracy or reliability of internet-based self-sampling screening remained a concern for many participants [32], for which, investigators should do efforts to figure out solutions for public education on cervical cancer prevention. Using PCR-based HPV assay is the key to ensure the sensitivity of self-collected samples [8]. In this study, we used Cobas 4800 and SeqHPV HPV testing assays, both were validated to work well with self-collected samples in multi-sectional control trials [33, 34].

There was only 58.7% of hrHPV-positive women returned to PUSH for colposcopy (the positive management rate, PMR) in this study. However, the lower PMR in this study cannot deny the potentiality of online screening in positive management because we did not require the participants to visit PUSH for positive management. Instead, we instructed them to search medical cares at any qualified medical facilities nearby them. PMR was not the key purpose of our study, but it is a key of cervical cancer control and need to be further studied.

Our study has several limits. Firstly, the social background survey just covered a limited items and the sample size was small since not all women participating in the screening responded or completely responded to the questionnaires. However, the results from the social background survey do provide us with an encouraging indication that promising application of online screening with further surveys encompassing a broader range of social elements and larger sample sizes are needed to provide robust evidence for the effectiveness of online screening. Secondly, we did not integrate a module into the website to facilitate women who tested hrHPV positive to schedule appointments for positive management services at hospitals, thus we could not provide evidence regarding whether online cervical cancer screening can increase the rate of positive diagnoses, which is vital to eliminate cervical cancer through screening. Finally, the constraint of internet facilitated screening may lie on potential exclusion of illiterate women or women without internet access at that time, which is no longer such constraint as internet is now a part of the human life.

## Conclusion

Based on the outcomes from our study, we concluded that (1) online screening is reliable in terms of self-registration, self-sampling, and self-ordering for specimen transportation; (2) online screening is suitable to women at all ages, regardless the variabilities in social elements, and (3) online screening facilitates cervical cancer screening to reach the women who have less accessibility to medical resources. Based on those concluded points, internet-facilitation is potentially an effective way to increase the screening coverage and avoid unnecessary screening repeat. All of that must be based on a professionally designed website.

## Data Availability

The datasets used and/or analyzed during the current study are available from the corresponding author upon reasonable request.
